# Policy-practice gaps and bottlenecks to postpartum family planning scale-up in the United Republic of Tanzania

**DOI:** 10.3389/fgwh.2026.1875076

**Published:** 2026-10-13

**Authors:** Charles Basil Kafaiya, Stella P. Kilima, Mary T. Mayige, Kijakazi O. Mashoto

**Affiliations:** National Institute for Medical Research, Headquarters, Dodoma, Tanzania

**Keywords:** barriers, bottlenecks, contraception, family planning, PPFP, Tanzania

## Abstract

**Background:**

Unintended and closely spaced births remain important public health concerns. Postpartum family planning provides an opportunity to prevent unintended pregnancies and improve maternal and child health outcomes. However, evidence on the implementation bottlenecks affecting PPFP scale-up in Tanzania remains limited.

**Aim:**

This study assessed implementation bottlenecks affecting the uptake, scale-up and sustainability of PPFP services in Tanzania and identified and prioritized potential actions to address them.

**Methods:**

A cross-sectional study employing a participatory mixed-methods approach was conducted using an adapted WHO PPFP bottleneck assessment tool, stakeholder survey, in-depth interviews, routine health information and population-based survey data, and review of national policies and guidelines. The online stakeholder survey included 477 invited participants, of whom 424 responded (88.9%). In-depth interviews were conducted with 34 stakeholders from regions representing high and low PPFP uptake. Findings from the different data sources were triangulated and presented during a stakeholder consensus meeting, where bottlenecks were ranked and prioritized and corresponding actions were identified.

**Results:**

Routine and survey data indicated substantial variation in PPFP uptake and an increase in postpartum FP counselling over time, but counselling did not consistently translate into PPFP uptake. The assessment identified gaps in provider capacity, motivation, financing, access to and awareness of updated policies and guidelines, referral systems, equitable access, and the scale-up of innovations. Policy reviews further identified gaps between national guidance and implementation, including limitations in dissemination, monitoring, and alignment with WHO recommendations. The consensus process prioritized actions addressing provider motivation, financing, dissemination of updated guidance and job aids, referral systems, equitable access, innovation scale-up, and provider capacity.

**Conclusion:**

Tanzania has a supportive policy environment for PPFP, but implementation remains constrained by interconnected health-system, provider, and service-delivery barriers. Addressing these bottlenecks through coordinated investments in financing, provider capacity and motivation, guideline dissemination, referral systems, equitable access, and scale-up of effective innovations is essential to strengthen PPFP implementation and uptake.

## Background

Unintended and closely spaced births remain significant public health concerns, contributing to increased maternal, neonatal, and child morbidity and mortality (1, 2). Short birth intervals are defined as conception occurring less than 24 months after a previous delivery, are associated with increased maternal and child health outcomes, including increased risk of preterm birth, low birth weight, small for gestational age infants, chronic undernutrition, stunted growth, and higher rates of child mortality (3, 4). Children born within 24 months of a previous birth have approximately a 60% higher risk of infant mortality compared with those born after intervals of more than 24 months (5).

According to the World Health Organization (WHO) (2022), the postpartum period begins immediately after the delivery of the placenta and extends up to 42 days (6). However, WHO also emphasises the importance of continued care during the first six months postpartum. This is a very important period to continue monitoring the mother and baby on issues of breastfeeding, family planning (FP), immunisations, HIV testing or treatment, and management of any complications that may arise (6).

Globally, more than 90% of women express a desire to delay or avoid pregnancy for at least one year after childbirth (7). An interval of at least 24 months before attempting the next pregnancy reduces the risk of adverse maternal, perinatal, and infant health outcomes (8). Therefore, the practice of post-partum family planning (PPFP) is a critical intervention with the potential to reduce maternal and child mortality by 30% and 10%, respectively (9). In addition, counselling for FP as part of care provided during childbirth increases postpartum contraceptive use and is likely to reduce both unintended pregnancies and closely spaced pregnancies (7). Despite this evidence, analysis of 22 Demographic and Health Surveys conducted between 2005 and 2012 in 21 low- and middle-income countries found that 61% of postpartum women had an unmet need for family planning (9, 10). Barriers to PPFP uptake include sociocultural and gender norms influencing postnatal practices, timing of return to menses and sexual activity, breastfeeding practices and misconceptions about lactational amenorrhoea, and lack of access to contraceptive services (11–13).

Proactively offering family planning as part of care during and immediately after childbirth is often referred to as immediate postpartum family planning. This is a common approach to address gaps in access to services. WHO recommends the provision of FP counselling and services throughout the continuum of maternal and child health care, including during antenatal care (6, 14, 15). Each visit to a health professional offers a unique opportunity to screen, counsel, and offer family planning services. Yet each opportunity requires deliberate attention to organize services, update policies and provider practices, and mobilize resources for successful implementation. Facility-based childbirth services offer an ideal platform to provide women and their partners with family planning information and services.

Despite efforts to scale up evidence-based, WHO-recommended family planning interventions, PPFP in sub-Saharan Africa (SSA) remains slow. A systematic review and meta-analysis of 33 studies from low-income countries in SSA estimated the pooled prevalence of postpartum contraceptive use at 37.4% (95% CI: 31.35–43.48%), indicating suboptimal utilization compared with global recommendations (16). Key factors associated with low PPFP uptake include limited knowledge and awareness, inadequate counselling, sociocultural norms, attitudes and beliefs, and health-system and provider-related barriers.

In Tanzania, while national stakeholders and policymakers demonstrate a strong commitment to PPFP, scaling up and sustaining these services has been challenging due to various bottlenecks at the community and health system levels. In this study, PPFP scale-up refers to the expansion and sustained delivery of evidence-based PPFP services across health facilities, service-delivery platforms, and geographic settings, supported by adequate human resources, financing, commodities, policies, systems, and implementation capacity. Thus, this study was conducted to assess country-specific barriers to PPFP uptake, scale-up and to inform strategies for improving service delivery and policy implementation in line with WHO guidelines.

## Material and methods

### Study setting

This study was conducted in Mainland Tanzania and Zanzibar, covering selected regions with varying levels of PPFP uptake based on District Health Information System (DHIS2) data. In Mainland Tanzania, Mara, Iringa, Arusha, and Kilimanjaro represented regions with high PPFP uptake, whereas Ruvuma, Njombe, Mtwara, and Pwani represented regions with low PPFP uptake. In Zanzibar, Kaskazini Pemba and Kaskazini Unguja were included among the regions with low PPFP uptake. No region in Zanzibar met the criteria for classification as a high-uptake region based on the available data; therefore, only low-uptake regions were included from Zanzibar. While the online structured questionnaire was shared across all regions of the United Republic of Tanzania (URT), in-depth interviews were conducted in these 10 purposively selected regions to capture perspectives from areas with contrasting levels of PPFP uptake.

### Study design

A cross-sectional study design which employed participatory mixed-methods to comprehensively assess PPFP implementation and identify bottlenecks affecting its uptake, scale-up and sustainability in Tanzania.

### Study population, sample size, sampling and response rate

The study population comprised healthcare workers and other stakeholders involved in the governance, management, and provision of FP and reproductive and child health (RCH) services. For the online stakeholder survey, approximately 14 respondents per region were targeted, including Regional and District Medical Officers, RCH/FP coordinators, pharmacists, and service providers working across health facilities and the community. Based on the number of regions, the estimated sample size was 434; allowing for 10% non-response, the target was increased to 477. Participants were purposively selected based on their professional roles and involvement in FP and RCH service delivery or management. Of the 477 stakeholders invited, 424 completed the questionnaire, giving a response rate of 88.9%.

The majority of respondents were health professionals 275 (88.4%), with 398 (93.9%) working in public institutions and 26 (6.1%) in private or non-governmental organization (NGO) settings. Most respondents worked at the council 195 (46%) and community 149 (35.1%) levels. Although the assessment captured perspectives from stakeholders across different levels of the health system, private facilities and NGOs were underrepresented. The distribution of respondents by institution, position, and working level is presented in Table 1.

**Table 1:** Profile of online respondents (*N* = 424).

Institution/Organization	Number	%
Public	398	93.9
Other (Private, NGO)	26	6.1
Position		
Policy	6	1.4
Program Manager	23	5.4
Researcher/M&E	0	0.0
Health professional	275	88.4
Other	20	4.7
Working level		
National	3	0.7
Region	27	6.4
Council	195	46.0
Community	149	35.1
Other	50	11.8

To obtain in-depth insights into challenges affecting the scale-up and implementation of PPFP, 34 in-depth interviews were conducted (Table 2). Ten regions were purposively selected to represent settings with high and low levels of PPFP uptake. Four high-uptake regions (Mara, Iringa, Arusha, and Kilimanjaro) and six low-uptake regions (Ruvuma, Njombe, Mtwara, Pwani, Kaskazini Pemba, and Kaskazini Unguja) were identified based on 2023 DHIS2 data for Tanzania Mainland, and through consultation with the national maternal and child health coordinator in Zanzibar where uptake of PPFP in all five regions is low.

**Table 2:** Interviewed individuals.

Informant	Interviewed			Planned
	Mainland	Zanzibar	All	
Director (RMNCAH)	1	1	2	2
Regional Coordinator RCH/FP	6	1	7	10
District coordinator RCH/FP	8	2	10	10
Health provider RCH/FP	13	2	15	30
Total	28	6	34	52

In each selected region, one council was selected from the respective high- or low-uptake stratum. Within each selected council, one district hospital, one health centre, and one dispensary were selected for inclusion. At each selected health facility in the respective district the plan was to interview either facility in-charge or service provider responsible for RCH and/or FP services, depending on who was available at the time of data collection. Hence the sample size at the health facility level was 30 people. The saturation point was reached after the interviews of 15 service providers/in charge of health facilities. At district and regional level, the plan was to interview 20 RCH coordinators (one per region and one per district). However, we managed to interview 17 out of the planned 20 district and regional RCH coordinators. The remaining three were not available at the time of data collection. At the national level, we interviewed two RMNCAH directors (one from Mainland and one from Zanzibar). This purposive sampling strategy was used to capture perspectives from stakeholders involved at different levels of PPFP governance, management, and service delivery.

### Recruitment and data collection procedures

Data were collected using structured WHO PPFP assessment tools adapted to the Tanzanian context to ensure cultural relevance and alignment with national policies. The adaptation focused on contextualizing the content and terminology of the WHO tools to reflect the Tanzanian PPFP policy and service-delivery context while retaining the original assessment framework. The tools were translated into Kiswahili to facilitate comprehension and consistent administration among participants. Findings from the assessment informed a stakeholder consensus meeting, during which key bottlenecks affecting PPFP uptake were reviewed and prioritized, and strategies to address the identified bottlenecks were discussed.

Regional RCH/FP coordinators shared the online structured questionnaire to eligible healthcare workers and relevant Civil Society Organizations (CSOs) and Community-Based Organizations (CBOs) involved in RCH and FP activities within their respective regions. Recruitment and data collection for the online survey commenced on 21 November 2024 and concluded on 25 December 2024. In-depth interviews were subsequently conducted with purposively selected stakeholders to obtain detailed perspectives on challenges affecting the scale-up and implementation of PPFP. The in-depth interviews and stakeholder consensus workshop were completed by 25 February 2025.

Quantitative and qualitative data were collected using complementary approaches and triangulated to provide a comprehensive understanding of barriers across the PPFP implementation pathway. The resulting findings informed a structured stakeholder consensus meeting, convened by the Ministry of Health, and a prioritization process through which the identified bottlenecks were reviewed, validated, ranked, and matched with potential strategies for improvement. Participants included policymakers and decision-makers, programme managers and implementers, health managers, healthcare providers, and representatives of relevant organizations.

### Data collection tools

#### Structured questionnaire

An adapted WHO PPFP bottleneck analysis tool was used to collect quantitative data through the online stakeholder survey (17). The questionnaire comprised eight categories covering 37 items (Table 3) and was designed to assess key factors influencing PPFP implementation and uptake. Responses to the 37 items were scored using a five-point Likert scale, ranging from 1 (fully agree) to 5 (fully disagree). For each assessment category, the mean bottleneck score was calculated by summing the item scores and dividing by the number of items assessed. Higher mean scores indicated greater perceived barriers to PPFP implementation. The scores were interpreted as not a bottleneck (1.00–1.99), minor bottleneck (2.00–2.99), important bottleneck (3.00–3.99), and very important bottleneck (4.00–5.00), and items were ranked according to their mean scores. No respondents selected the “Don't know” option for any assessed item; therefore, it was not included in the mean score calculation. The responses were used to quantify perceived bottlenecks across the assessed domains and provided a structured basis for subsequent analysis and comparison of PPFP implementation challenges.

**Table 3:** Number of assessed items per bottleneck analysis category.

Category	Number of question items
1. Policy implementation status	1
2. Governance	4
3. Financing	6
4. People	6
5. Information	5
6. Medical and technology	3
7. Service delivery	6
8. Human resources	6

#### In-depth interview guide

An in-depth interview guide was developed based on the same eight categories assessed in the adapted WHO PPFP bottleneck analysis tool (17). The guide included open-ended questions and probing questions designed to elicit detailed stakeholder perspectives on the identified challenges, enabling factors, and successes related to PPFP implementation. The interviews complemented the structured questionnaire by providing contextual and explanatory insights into the factors underlying the identified bottlenecks and facilitators.

#### Checklist for desk review of national data and policy alignment assessment

An adapted WHO checklist was used to assess the alignment of national policies and to review routinely reported data relevant to PPFP. Secondary data from the DHIS2 for the period 2020–2023 were extracted to examine trends in the provision of FP counselling services and PPFP uptake. These findings were compared with data from the 2022 Tanzania Demographic and Health Survey (TDHS) to assess consistency in observed trends and identify potential differences or disparities between routine health information and survey-based estimates. Relevant national policies, guidelines, strategies, and other programmatic documents were identified through targeted searches of electronic databases and search engines, including Google Scholar and PubMed, as well as other relevant sources. The identified documents were reviewed to assess the extent to which national policies and guidance were aligned with relevant international and regional recommendations for PPFP. Key documents reviewed included the Third National Five-Year Development Plan; Tanzania Development Vision 2025; Zanzibar Vision 2020; Zanzibar Family Planning Costed Implementation Plan (2018–2022); Zanzibar Health Sector Strategic Plan IV; Tanzania Health Sector Strategic Plans IV and V; One Plans II and III; the National Family Planning Costed Implementation Plan II (NFPCIP II, 2019–2023); the National Family Planning Guidelines and Standards; the National Health Policy; National Guidelines for Antenatal Care for Service Providers; the Tanzania Prevention of Mother-to-Child Transmission (PMTCT) Pocket Guide; and the Zanzibar Strategy for Growth and Reduction of Poverty III (MKUZA III) (18–30).

These documents served complementary roles within the national health and development framework. The national development visions and five-year development plans provide the overarching development priorities and strategic direction for health and population programmes. The National Health Policy and Health Sector Strategic Plans translate these broader priorities into health-sector objectives, priorities, and implementation directions. The *One Plans* provide a strategic framework for strengthening reproductive, maternal, newborn, child, and adolescent health and nutrition services, including the integration of family planning across the continuum of care. The FP Costed Implementation Plans outline priority interventions, implementation approaches, and resource requirements for strengthening FP services, while the National Family Planning Guidelines and Standards and other clinical guidance documents, including antenatal care (ANC) and PMTCT guidance, provide more specific recommendations for the delivery and integration of services at facility and community levels. The Zanzibar-specific strategies and plans were reviewed to capture the policy and implementation context within Zanzibar.

### Researcher positionality and reflexivity

The qualitative data were collected and analysed by researchers with backgrounds in sociology and public health, most of whom work in family planning, reproductive and child health, and health systems research. Researchers involved in data collection were not directly responsible for the routine performance assessment of the participants. To minimize potential researcher influence, interviewers used a standardized semi-structured interview guide and maintained a neutral approach throughout the interviews. During analysis, researchers independently reviewed and coded the data, followed by regular discussions to compare interpretations, resolve discrepancies, and reach consensus on emerging themes. Reflexive discussions were also undertaken to consider how the researchers’ professional backgrounds and familiarity with the PPFP context might have influenced data interpretation.

### Integration and triangulation of findings

Findings from the different data sources were integrated using a triangulation approach. Quantitative bottleneck scores from the online stakeholder survey were examined alongside themes emerging from the in-depth interviews and evidence from the DHIS2, TDHS, and policy and document reviews. The quantitative findings provided an indication of the relative magnitude of perceived implementation bottlenecks, while qualitative findings were used to provide contextual explanations and identify experiences, enabling factors, and implementation challenges that could not be captured through the structured questionnaire. Evidence from routine and survey data was used to contextualize observed patterns in PPFP uptake, while the policy review was used to assess whether the identified implementation challenges were reflected in, or potentially related to, the existing policy and programmatic environment. Taken together, these complementary sources provided a broader evidence base for the subsequent stakeholder validation and prioritization of PPFP bottlenecks.

### Analysis of the online stakeholder survey and in-depth interviews

Data from the online stakeholder survey were analyzed descriptively and summarized using mean scores, disaggregated by respondents’ level of the health system. For each of the eight categories, the mean score was calculated by dividing the sum of the scores assigned to the individual items by the number of items comprising that category. Higher mean scores indicated greater perceived bottlenecks or implementation challenges within the corresponding category. Respondents who selected “don't know” were treated as the reference response and were not assigned a Likert-scale score. Given the small number of survey respondents from Zanzibar (*n* = 4), comparative analyses between Tanzania Mainland and Zanzibar were not performed.

For the in-depth interviews, audio recordings were transcribed verbatim by two experienced research assistants. The transcripts were reviewed against field notes and validated by cross-checking them with the original audio recordings to ensure accuracy and completeness. The verified transcripts were imported into Microsoft Excel for manual thematic analysis. A codebook was developed based on the study objectives and the domains of the adapted WHO PPFP bottleneck assessment tool, while allowing additional codes to emerge inductively from the data. Transcripts were systematically coded and organized into categories. Themes and sub-themes were subsequently developed from the coded data, capturing stakeholders’ perspectives on the challenges, enabling factors, and successes related to PPFP implementation. Coding discrepancies and emerging themes were discussed and resolved through consensus between the researchers. The final themes were reviewed against the original transcripts to ensure consistency and credibility of the findings.

### Stakeholder consensus and prioritization

A stakeholder consensus meeting was conducted to review and validate findings from the online survey, in-depth interviews, routine data, and policy review. Stakeholders from high- and low-PPFP-uptake regions, together with implementing partners from selected PPFP programmes, participated in the meeting and shared their perspectives and implementation experiences. Following presentation of the assessment findings, participants were divided into three groups: Group 1 focused on bottlenecks related to governance and finance; Group 2 focused on people and information; and Group 3 focused on medicines/technology, service delivery, and human resources. Participants independently scored each assessed bottleneck item, and mean scores were calculated and ranked to determine their relative importance. Based on the consensus workshop scores, items were classified as very important, important, minor, or not bottlenecks. Each group presented its top five bottlenecks to the larger group and discussed the types of factors considered, their ranking, and the rationale for selecting the most important bottlenecks.

Six key questions guided the prioritization and development of actionable recommendations: (a) how big of a problem is this factor in preventing scale-up of PPFP; (b) if it was addressed, would we see improvements in the scale and quality of PPFP; (c) is this a problem preventing nationwide scale-up of PPFP; (d) how urgently does this bottleneck need to be solved; (e) how many other bottlenecks does this problem cause; and (f) can this bottleneck actually be resolved with careful planning and resource allocation. The ranked findings were then discussed in groups to examine the underlying causes of prioritized bottlenecks and identify feasible solutions. This process enabled stakeholders to validate and prioritize the key PPFP bottlenecks and subsequently develop an action plan to address the identified gaps.

From each selected region, up to two participants represented their region during the stakeholder consensus and prioritization process. A single national set of priorities was produced; however, given the variations observed across different levels of the health system, this approach may have obscured region-specific bottlenecks and implementation needs. To address this, workshop participants were trained on the same ranking and prioritization approach and were requested to apply it in their respective regions to generate region-specific priorities and actions to address the most important bottlenecks identified.

### Ethical considerations

Ethical approval for this study was obtained from the National Research Ethics Committee (NatHREC) under the approval number NIMR/HQ/R.8a/Vol. IX/4756. These study procedures were demonstrated by the development of ethical guidelines and regulations. Written informed consent was obtained from all participants before data collection, and confidentiality and anonymity were maintained throughout the study; no personal identifies were included in the analysis or reporting of the findings. No minors were included in this study.

## Results

### Policy environment, implementation and dissemination of PPFP guidance

Tanzania has a supportive policy environment for FP, with PPFP incorporated into national strategies and identified as a high-impact intervention for improving maternal, newborn, child and adolescent health. The country's commitment to family planning is reflected in several key national and subnational policy and strategic documents, including the Third National Five-Year Development Plan, the National Health Policy, Health Sector Strategic Plans IV and V, One Plan II, the National Family Planning Costed Implementation Plan II (NFPCIP II), the National Family Planning Guidelines and Standards, the National Guidelines for Antenatal Care for Service Providers, and relevant Zanzibar-specific family planning, health-sector, and development strategies (18, 21–23, 26, 28–32) The country has also launched campaigns to raise awareness of the benefits of FP and reduce stigma and misconceptions surrounding contraceptive use. Family planning services have been integrated into Maternal and Child Health care since 1974, and the country has prioritised PPFP as one of the high-impact practices to accelerate improvement of maternal, newborn, child and adolescent health (33). However, the policy review identified important gaps between policy provisions and implementation. For example, although national guidance promotes integration of FP services into ANC, postnatal care (PNC), immunization and post-abortion care, implementation varies across facilities because of differences in health-system capacity, availability of trained providers and other resources. The policy review also identified gaps in PPFP-specific monitoring indicators and limitations in the alignment of some national guidance with WHO recommendations.

The *One Plan II* prioritizes male involvement in sexual and reproductive health; however, gaps remain in healthcare providers’ awareness and understanding of meaningful male participation while maintaining women's autonomy in FP decision-making (28). The current *One Plan III (2021–2025)* further emphasizes improving the quality of FP information and counselling during ANC and breastfeeding counselling sessions, scaling up PPFP services, strengthening FP integration across health facilities, expanding partnerships with private and NGO-owned facilities, and increasing the capacity of community health workers to provide FP counselling and approved methods at community level (24). It also promotes greater community participation across the RMNCAH continuum of care, including ANC, labour and delivery, and postpartum care. Despite these policy commitments, the review identified gaps between policy provisions and implementation, including limited PPFP-specific monitoring indicators, inadequate mechanisms for measuring integration across high-volume services, and incomplete alignment of some national guidance with WHO recommendations. For example, national guidance requires FP integration into ANC, PNC, immunization and post-abortion care, but implementation varies across facilities according to health-system capacity, availability of trained providers and other resources.

The policy review also identified operational gaps in the alignment of national guidance with international recommendations, including the need to clarify immediate PPFP method options, strengthen the role of all antenatal and maternity care providers in PPFP provision, and define the role of community health workers in promoting PPFP. These policy-level findings were reinforced by the in-depth interviews, which highlighted challenges in the dissemination, accessibility and routine use of PPFP guidelines, particularly at lower levels of the health system. Some participants reported inadequate availability of guideline copies and limited opportunities for orientation and refresher training:

“There are no enough copies of PPFP guidelines; only two copies were available to the two health workers who got trained on PPFP. That is a challenge because we have many health centres. The Ministry of Health should distribute the guidelines according to the number of health facilities and not only to those who received training.” (KII-HCP, Iringa District).

However, perspectives differed across levels of the health system. While frontline participants reported limited access to guidelines and training, a national-level participant indicated that guidelines were available at facilities:

“When we conduct training, we ensure the service providers leave with those guidelines. All facilities offering family planning services already have the family planning guidelines with them.” (National-Level Participant, Tanzania Mainland).

This divergence suggests that the challenge may extend beyond the formal availability of guidelines to their consistent dissemination, accessibility, orientation and utilization at service-delivery level. This was further illustrated by reports of limited orientation and continuous training:

“We have not been enabled to disseminate family planning guidelines anymore; we have not conducted any training or orientation. This affects the quality-of-service delivery significantly because, to provide services, you need to know the aspects of operations of the service you are offering.” (KII, Njombe Regional Referral Hospital).

In Zanzibar, participants additionally identified the format and usability of the guidelines as barriers and expressed a preference for simplified tools such as flyers, posters and visual job aids. One regional level participant noted:

“The use of available FP guidelines is a challenge. The book is just too big. It would be more effective to have relevant information in the form of a flyer or poster.” (Regional-level participant, Kaskazini Unguja, Zanzibar).

Guidelines come from the MoH; training for these guidelines is rarely done. (Service Provider, Kaskazini Pemba, Zanzibar).

These findings were consistent with the views of participants in the consensus meeting, in which communication, knowledge and awareness were classified as a very important bottleneck (mean score 4.00), while guidelines and tools were classified as an important bottleneck (mean score 3.45). Guidance formulation and health promotion were also classified as important bottlenecks (mean score 3.00 each).

### Service integration and delivery platforms

Reproductive and Child Health (RCH) clinics serve as the primary delivery points for FP services, with FP counselling and services provided through ANC, PNC, immunization, HIV/AIDS care, and post-abortion care platforms. National guidance requires FP counselling and services to be provided alongside post-abortion care, including counselling for informed choice and method-specific counselling (32). However, the extent to which these integrated service-delivery opportunities translate into PPFP uptake varies across settings.

According to the national ANC guidelines, FP information is initiated at the fourth ANC visit, with subsequent visits focusing on dual protection for FP and HIV prevention during the postpartum period (34). This differs from WHO recommendations, which advocate providing information on different FP methods, including their benefits and side effects, at each ANC visit (15). At the population level, although more than 80% of childbirths occur in health facilities, only 3.3% and 19% of women were using a modern FP method at one and six months postpartum, respectively, according to the 2022 TDHS (27). PPFP use also varied by place of delivery, with women who delivered at home reporting lower use of modern PPFP methods than those who delivered in health facilities.

The routine data further demonstrated increasing provision of FP counselling within postpartum care. However, the absence of indicators tracking transitions from Lactating Amenorrhea (LAM) to other FP methods and the lack of routine measures of FP services received immediately after delivery, at one month, and at six months limited assessment of continuity of PPFP use. These data limitations constrained assessment of continuity of PPFP following counselling.

The participants in the consensus meeting and in-depth interviews provided additional evidence on factors affecting service integration and delivery. Service organization and scheduling (mean score 3.18) and referral systems (3.45) were classified as important bottlenecks. Interview participants similarly described limitations in the availability of trained providers and access to specific PPFP methods across facilities. These findings complement the routine and survey data by indicating that the presence of multiple service-delivery platforms does not necessarily ensure consistent access to comprehensive PPFP services.

### Alignment of FP guidance with regional and international policies

The policy environments in Tanzania are broadly supportive of PPFP implementation. However, several policy and operational gaps remain that hinder full alignment with the international and regional guidelines issued by the WHO (22). For instance, the national FP and standards need to be revised to include progestin-only methods and implants as options for immediate PPFP use. Job descriptions for service providers need to clearly articulate that all antenatal and maternity care providers have a role in postpartum family planning, and that it is not just the responsibility of a few trained providers. The role of community health workers in promoting postpartum family planning needs to be specified in the job aids.

### FP counselling and PPFP uptake

As per Tanzania mid-term assessment of NFCIP II, FP counselling during ANC visits has increased from 19% in 2020 to 132% in 2023; however, the uptake of PPFP services remains low (26). Currently, the integration of breastfeeding counselling with FP service provision has not contributed much to the attainment of the national goal of 60% FP uptake. The reported increase in FP counselling uptake in the labour wards and within 2–6 weeks after birth has not translated into increased uptake of FP services. Therefore, it is important to determine why the uptake of FP services remains low. Such information will help determine the quality of counselling, its effectiveness, women's reasons for not initiating any FP after counselling, and identify barriers that prevent women from adopting FP methods.

The report of the mid-term assessment of NFPCIP II (2019–2023) implementation revealed a steady increase in PPFP use, from 14.9% in 2019 to 45.8% in 2023 (26). However, the uptake of modern FP after abortion declined slightly from 82.9% in 2021 to 78.3% in 2023 (Figure 1). PPFP and post-abortion modern FP uptake varied across regions of Tanzania Mainland. The regions with high use of PPFP included Mara (73.6%), Iringa (72%), Arusha (67.8%), and Kilimanjaro (66.2%), while regions with low PPFP use were Ruvuma (8.4%), Mtwara (19.7%), Pwani (21.4%), and Njombe (19.1%). Among young women, regions with high PPFP uptake were Morogoro, Kigoma, Dar es Salaam and Mwanza, while regions with low PPFP uptake among young women were Songea, Singida, Njombe, and Pwani. The lowest uptake of modern FP post-abortion was reported in Njombe (43%), Kagera (51%), and Rukwa (59%).

**Figure 1:**
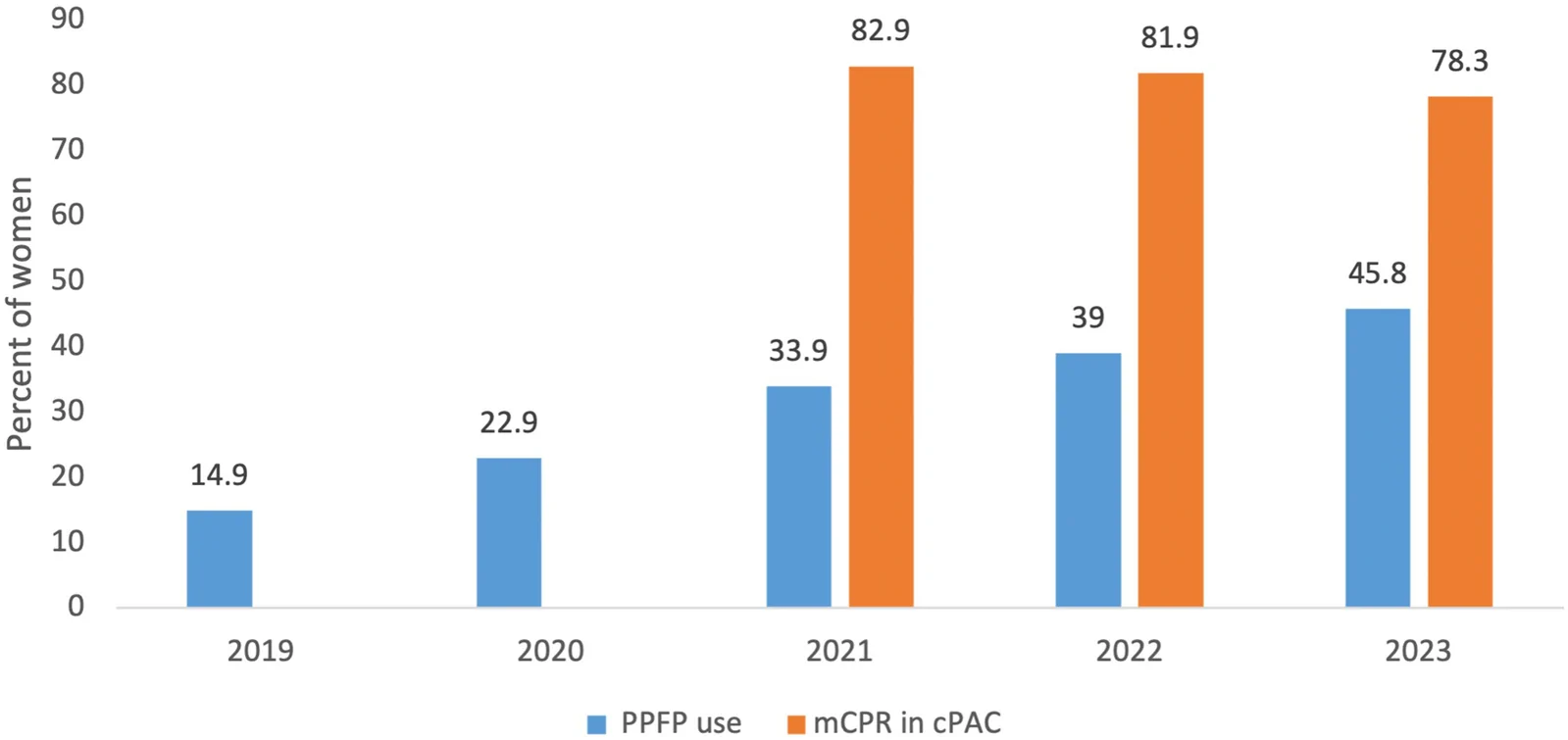
Use of PPFP and post-abortion modern FP.

A review and analysis of DHIS2 data from 2020 to 2023 revealed a substantial increase in the number of postpartum clients receiving FP counselling within 6 weeks after birth (Figure 2). However, the absence of an indicator to track transition from LAM to other FP methods, together with the lack of routine measures of FP services received immediately after delivery, at one month, and at six months, limited assessment of continuity of PPFP use.

**Figure 2:**
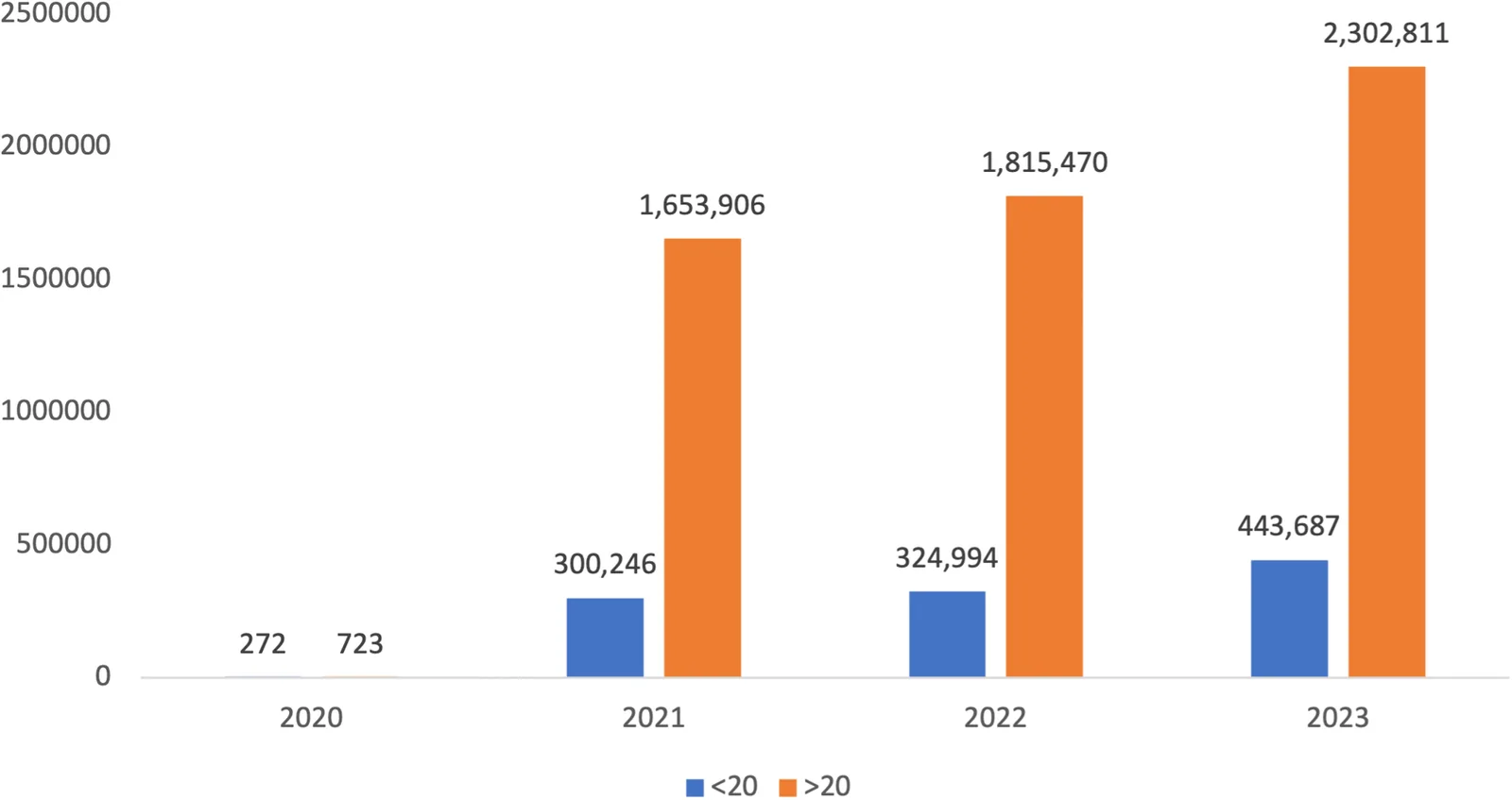
Number of PNC clients who received FP counselling 2–6 weeks postpartum.

The quantitative findings from Figures 1, 2 demonstrate a gap between increasing FP counselling coverage and actual PPFP uptake, alongside substantial regional variation in uptake. Findings from the in-depth interviews provided contextual explanations for these patterns, particularly regarding healthcare provider capacity and the availability of a broad range of contraceptive methods. Participants reported that long-term and permanent methods, such as IUD insertion, were available in only a limited number of facilities and that trained providers were insufficient. Consequently, providers often relied primarily on short-term methods and, in some facilities, offered only a limited number of implants. For example:

“Long-term and permanent methods are also a challenge because there are very few facilities that offer them.” (KII-HCP, Mara).

Similarly:

“For example, with IUD insertion, there are very few trained staff, so they mostly provide short-term methods, and they also offer a few implants.” (KII-HCP, Pwani).

Limited provider capacity was also reported at facility level:

“The capacity to deliver PPFP is very low, for example, here in Mji Mwema, only two people got trained on PPFP.” (KII-HCP, Songea).

These qualitative findings provide contextual explanations for the gap between counselling coverage and PPFP uptake observed in the quantitative data. They suggest that counselling may not translate into uptake when women have limited access to providers able to offer their preferred methods, particularly long-term and permanent methods. This interpretation was consistent with the participants in the consensus meeting, in which capacity was classified as an important bottleneck (mean score 3.27). Referral systems (mean score 3.45) and service organization and scheduling (mean score 3.18) were also classified as important bottlenecks. Together, these findings indicate that provider capacity, method availability, and health-system arrangements may influence the translation of counselling opportunities into PPFP uptake.

The qualitative findings also highlighted that counselling itself does not necessarily result in contraceptive adoption, as the final decision remains with the woman. As one healthcare worker from Iringa explained:

“We provide family planning and breastfeeding counselling to many women who deliver at our facility. However, the decision to use a particular PPFP method rest with the woman.” (KII, HCW, Iringa).

This finding provides context for interpreting the increase in counselling shown in Figure 2 and the relatively lower uptake shown in Figure 1, emphasizing that counselling coverage should not be equated with contraceptive adoption.

In contrast, experiences from regions where NGOs were involved in FP scale-up suggested that additional technical capacity and community-based demand-creation approaches could facilitate service expansion. An NGO representative from Kilimanjaro reported:

“Our NGO has a project called scale up family planning, which is implemented in eight regions of Tanzania. We have all the knowledge and capacity needed for FP scale-up. For demand creation, we use HCWs and advertising vehicles.” (KII, NGO representative, Kilimanjaro).

### Stakeholder perspectives on factors inhibiting PPFP scale-up

More than 80% of the stakeholder survey respondents were health workers at council and community levels, while 1.4% were policymakers and 10.1% were programme managers and implementers. Disaggregated analysis showed variation in perceived bottlenecks by level of the health system. At the national level, budgeting, insurance, and spending were identified as the most important bottlenecks, while networks and motivation were classified as important. At the regional level, budgeting, spending, and motivation were identified as important bottlenecks (Table 4).

**Table 4:** Most important bottlenecks for PPFP uptake in Tanzania.

PPFP bottleneck domain	National	Region	Council	Community	Other	All
Implementation status	2.33	2,41	1.55	1.53	1.76	1.63
Governance/Accountability	2.00	2.30	1.63	1.68	1.66	1.70
Financing/Budgeting	4.33	3.26	2.43	2.44	2.54	2.51
Financing/Insurance	5.00	2.81	2.60	2.52	2.80	2.63
Financing/Spending	4.33	3.07	2.35	2.46	2.56	2.48
People/Networks	3.33	2.89	2.01	2.20	2.04	2.15
Human Resources/Motivation	3.33	3.33	2.55	2.68	3.14	2.72

These findings were consistent with evidence from the policy review and in-depth interviews, which identified resource availability, financing, provider motivation, and health-system capacity as challenges affecting PPFP implementation. The convergence of findings across the stakeholder survey, qualitative interviews, and policy review indicates that financial and health-system factors were perceived as important constraints to PPFP scale-up, particularly at national and regional levels.

The qualitative findings further contextualized these stakeholder perceptions, with participants describing weaknesses in health-system support for PPFP delivery, including inadequate clinical supervision, ineffective referral mechanisms between health facility units, limited dissemination and adoption of PPFP innovations, and insufficient government and donor financing for large-scale implementation. Participants also reported limited communication, knowledge, and awareness of PPFP guidance among service providers. In Zanzibar, participants noted that although PPFP guidance had been updated, PPFP use remained low, while on the mainland, participants highlighted the need for updated and widely disseminated guidance. These findings were consistent with the participants in the consensus meeting, particularly the high ratings for budgeting, spending, motivation, insurance, and networks at national and regional levels, demonstrating convergence between the stakeholder survey and qualitative evidence.

These challenges were also reflected in the experiences of interview participants. One participant described the effect of limited health-system support on PPFP implementation:

“Financing from the government is not available, only donors contribute to the provision and dissemination of information on PPFP/FP.” (KII-Regional level participant, Kaskazini Pemba, Zanzibar)

Another participant highlighted how limited human-resource capacity constrained the delivery and expansion of PPFP services:

“Capacity is compromised because of limited human resources, as there are not enough HCWs to sufficiently provide PPFP. With only two HCWs, it is difficult to cover ANC, RCH, and high-risk services while also counselling women on long-term FP methods such as IUCD.” (KII-Regional level participant, Kaskazini Unguja, Zanzibar)

### Sociocultural and community-level factors influencing PPFP uptake

Stakeholders and interview participants also described sociocultural and community-level factors that may influence the uptake of PPFP. These included misconceptions and concerns regarding the safety and side effects of contraceptive methods, as well as beliefs and attitudes that may discourage contraceptive use.

One participant described how misconceptions and beliefs about the postpartum period influenced women’s decisions to use FP:

“Women believe that after giving birth, they are not allowed to have sex with their husbands, so they tend not to use FP until they start having sex again.” (KII-HCP, Ruvuma)

Participants also highlighted the influence of partners and family members on women's family planning decision-making. This was illustrated by one participant who noted:

“Patriarchy is a challenge; she has to get consent from her husband. Even their mothers-in-law have influence as well. You find someone saying, ‘I’m afraid of my mother-in-law.” (KII-HCP, Kilimanjaro)

These factors were perceived to contribute to limited awareness and acceptance of PPFP services within some communities. These qualitative perspectives complemented findings from the participants in the consensus meeting, which identified limited knowledge and awareness as an important implementation challenge. Together, the findings suggest that barriers to PPFP implementation extend beyond health-system and provider-level constraints and may also involve community-level factors that influence awareness, acceptability, and decision-making regarding contraceptive use.

### PPFP bottlenecks and priority actions

Findings from the online stakeholder survey, in-depth interviews, routine data, and policy review indicated that multiple and interconnected bottlenecks could constrain PPFP uptake, scale-up, and sustainability. The stakeholder survey identified challenges related to financing, provider motivation and capacity, information and awareness, referral systems, and service organization, while the qualitative findings provided contextual explanations of how these challenges could affect access to and uptake of PPFP services and the capacity to expand and sustain their delivery. Routine and population-based data provided additional evidence on patterns of FP counselling and PPFP uptake, highlighting gaps between service contacts and actual uptake, while the policy review identified gaps in the dissemination, implementation, and alignment of PPFP guidance. Taken together, these findings indicated that addressing these bottlenecks is important for improving PPFP uptake, supporting its expansion, and sustaining service delivery across facilities and regions. The findings were subsequently presented during the stakeholder consensus meeting. Stakeholders from high- and low-PPFP-uptake regions, together with implementing partners from selected PPFP programmes, reviewed and discussed the findings. Table 5 presents the most important bottlenecks identified through this process, together with their mean scores and the actions agreed.

**Table 5:** The most important PPFP bottlenecks identified during consensus workshop.

	Bottleneck	Mean score	Identified solution
1.	Low motivation	4.27	Provision of monetary, and non-monetary incentives such as certificate of recognition, and incorporate PPFP into service providers’ work package
2.	Inadequate budget	4.09	Advocate for allocation of adequate domestic funds for postpartum and post-abortion FP and include postpartum and post-abortion FP in the Global Financing Facility (GFF) Investment Case
3.	Limited knowledge and awareness on updated/existing policies and guidelines	4.00	Disseminate updated PPFP policies, guidelines, and job aids through clear communication channels
4.	Suboptimal referral systems for PPFP	3.45	Incorporate PPFP referral guidance into general referral guidelines/protocols
5.	Inequitable access to PPFP	3.36	Review data regularly to ensure equitable allocation of budget.
6.	Innovation not rapidly scaled up or adopted	3.36	Use locally generated evidence to mobilize resources and influence political or institutional commitment to scale innovations to support PPFP scale-up.
7.	Inadequate capacity for provision of PPFP services and information	3.27	Train health workers, incorporate PPFP topic in pre-service training and equip health facilities with supplies and equipment needed for provision of PPFP

The triangulated findings identified low motivation as the highest-ranked bottleneck (mean score 4.27), followed by inadequate budget (4.09) and limited knowledge and awareness of updated/existing policies and guidelines (4.00). Stakeholders proposed monetary and non-monetary incentives for low motivation; advocacy for adequate domestic funding and inclusion of postpartum and post-abortion FP in the Global Financing Facility (GFF) Investment Case for inadequate budget; and dissemination of updated PPFP policies, guidelines, and job aids through clear communication channels for limited knowledge and awareness.

Other prioritized bottlenecks included suboptimal referral systems for PPFP (3.45), inequitable access to PPFP (3.36), innovation not rapidly scaled up or adopted (3.36), and inadequate capacity for provision of PPFP services and information (3.27). The corresponding actions included incorporating PPFP referral guidance into general referral guidelines and protocols, regularly reviewing data to support equitable budget allocation, using locally generated evidence to mobilize resources and influence political or institutional commitment to scale innovations, and training health workers, incorporating PPFP into pre-service training, and equipping facilities with the required supplies and equipment.

## Discussion

This study assessed PPFP uptake in Tanzania, identified bottlenecks affecting PPFP implementation and scale-up, and highlighted gaps in national policy alignment with WHO policy guidance. The findings demonstrate that the existence of supportive policies and increased contact with the health system do not necessarily translate into effective PPFP implementation or uptake. Rather, the findings point to a persistent gap between policy intent, service delivery, and utilization. These findings are consistent with earlier studies across sub-Saharan Africa that reported policy practice mismatches in family planning scale-up (35, 36).

Our study revealed that Tanzania's national FP guidelines and *One Plan III* prioritize PPFP integration into RMNCAH services. However, family planning counselling is still introduced late during ANC visits, unlike the WHO recommendations, which advise counselling at every visit. This is similar to findings from the study conducted in Ethiopia, where delays in counselling reduced early contraceptive uptake (37). Furthermore, dissemination of guidelines remains uneven, particularly in Zanzibar. This finding mirrors studies in Malawi and Nigeria that identified weak dissemination channels as a key barrier to implementing updated reproductive health policies, including Family Planning (38, 39). The convergence of the policy review and qualitative findings suggests that the challenge is not simply the absence of policies or guidelines, but gaps in their dissemination, accessibility, provider orientation, and application at service-delivery level. This distinction is important because strengthening the policy framework alone may have limited effect if frontline providers do not have adequate access to, knowledge of, and support to implement the guidance.

Evidence from PPFP initiatives in Tanzania, including USAID-supported programmes such as Boresha Afya, suggests that PPFP scale-up can be supported through provider training, mentorship, and integration across service-delivery points (40, 41). However, this assessment was conducted before the changes to U.S. foreign assistance in 2025. Subsequent disruptions in USAID-supported programming may affect the sustainability and future scale-up of PPFP and post-abortion family planning services, highlighting the importance of strengthening domestic financing and national capacity. Similar experiences in Rwanda and Nepal have shown that capacity-building and service integration across delivery points can support improved uptake (42, 43). In contrast, contexts that lacked task shifting and community-based engagement, such as certain regions in Africa, showed slower progress in scaling up PPFP (36). Evidence indicates that community-based education through CHWs, schools, and digital platforms plays a role in increasing awareness and acceptance of the interventions (44). In the present study, the contrast between settings with limited provider capacity and those supported by implementing partners further illustrates the importance of combining provider capacity-building with community-level demand creation. However, reliance on partner-supported activities may result in uneven implementation if such approaches are not incorporated into routine government systems.

Stakeholder perspectives emphasized human resource shortages, limited training opportunities, and high workloads as major challenges. These findings are similar to those from studies in Zambia and Uganda, where provider shortages constrained the availability of long-term methods such as IUDs (45, 46). In contrast, in regions where NGOs invested in staff training and commodities, scale-up was more successful, suggesting that donor-supported interventions can temporarily bridge capacity gaps but require stronger government ownership for sustainability (47). Our findings further suggest that provider capacity is closely linked to the gap between FP counselling and actual PPFP uptake. Although routine data showed increased counselling, qualitative findings indicated that limited numbers of trained providers and restricted availability of long-term and permanent methods constrained women's ability to obtain their preferred methods. Thus, increasing counselling coverage without simultaneously strengthening service readiness may not be sufficient to achieve substantial improvements in PPFP uptake.

This study also identified sociocultural barriers, including male partner dominance and misconceptions about contraceptive safety, that hinder PPFP uptake. These findings are consistent with research in Nigeria, which highlighted male approval as a determining factor for contraceptive use (48, 49), and in contrast to some Asian settings, such as Bangladesh, where women's autonomy played a greater role in decision-making (50). The persistence of myths and misconceptions further aligns with evidence from Uganda, underscoring the need for robust social behavioural change interventions to counter misinformation (51, 52). These findings also highlight the importance of balancing male engagement with women's autonomy in PPFP decision-making. Male involvement should therefore support informed and voluntary contraceptive choice rather than become a prerequisite for women's access to FP services. Effective male engagement also requires providers to have appropriate counselling skills and guidance to involve male partners constructively while safeguarding women's informed and voluntary contraceptive choices. Strengthening provider capacity in this area may help address the intersection between provider-level and sociocultural barriers to PPFP uptake.

Finally, the consensus process provided an opportunity to integrate evidence from the different data sources and translate the identified challenges into actionable priorities. The findings from the consensus meeting suggest that PPFP scale-up is influenced by interconnected health-system and service-delivery factors, rather than by a single barrier. This is consistent with a review conducted in sub-Saharan Africa that identified inadequate domestic funding as a key barrier to sustainability (53). In contrast, settings with sustained budget allocation, such as Ethiopia's Health Extension Program, showed greater resilience in scaling up PPFP (54). These findings imply that sustainable PPFP scale-up in Tanzania will require coordinated interventions that address financing, human resource capacity and motivation, access to and use of guidelines, referral systems, and community engagement. PPFP responsibilities should be integrated into the roles of all antenatal and maternity care providers rather than being limited to a few trained staff. Community health workers should also be supported with clear guidance and job aids to promote PPFP awareness and facilitate appropriate referral. Strengthening referral systems, equitable resource allocation, and the scale-up of effective innovations will further support improved PPFP implementation and uptake. Such a coordinated approach may help bridge the gap between policy commitment and effective implementation of PPFP services.

The marked differences in perceived bottlenecks across levels of the health system suggest that actors’ vantage points strongly shape their priorities. National and regional respondents emphasized financing and budgeting, reflecting their direct involvement in resource mobilization, allocation, and donor negotiations. In contrast, council and community respondents rated financing as less critical, likely because they experience its effects indirectly and are more immediately constrained by staffing shortages, commodity availability, and supervision challenges. This divergence underscores a disconnect between macro-level policy concerns and frontline implementation realities, highlighting the importance of multi-level dialogue and participatory planning mechanisms to align priorities and foster coordinated solutions.

### Limitations

This study has several limitations. First, the stakeholder survey and in-depth interviews primarily captured perspectives of providers, programme managers, and health-system stakeholders. Women using or not using PPFP services and their partners were not directly included; therefore, findings on preferences, decision-making, method acceptability, counselling quality, and other demand-side factors are largely based on stakeholder perceptions and secondary data. This is particularly relevant to the gap between counselling coverage and actual PPFP uptake. Second, purposive selection of participants and regions, including only low-uptake regions in Zanzibar, limits generalizability and direct comparison across settings. Third, online survey administration may have limited participation among stakeholders with poor internet access. Fourth, differences between DHIS2 and TDHS data and limited PPFP-specific indicators constrained assessment of continuity of contraceptive use after counselling. Finally, although findings from the different sources were triangulated and subsequently reviewed during the stakeholder consensus process, the qualitative findings and stakeholder scores may still be influenced by participants’ experiences and perceptions. These limitations should be considered when interpreting the findings and their applicability to PPFP scale-up and sustainability in other settings.

## Conclusion

This study highlights that, despite Tanzania's supportive policy environment and efforts to integrate PPFP within routine maternal and child health services, important implementation challenges remain. Effective PPFP scale-up will require translating policy commitments into consistent practice through improved dissemination and use of guidelines, strengthened provider capacity and motivation, sustainable financing, functional referral systems, equitable access to services, and the adoption and scale-up of effective innovations. Addressing these interconnected health-system and service-delivery factors through coordinated action will be important for improving PPFP implementation and uptake in Tanzania

## Data Availability

The original contributions presented in the study are included in the article/Supplementary Material, further inquiries can be directed to the corresponding author.
